# Soil enzymatic activity data over eight years at the EFELE site, a long-term field experiment on effects of organic waste products and tillage practices

**DOI:** 10.1016/j.dib.2021.106959

**Published:** 2021-03-18

**Authors:** Nathalie Cheviron, Issifou Amadou, Virginie Grondin, Christelle Marrauld, Christian Mougin, Thierry Morvan

**Affiliations:** a UMR ECOSYS, Platform Biochem-Env, INRAE^1^, AgroParistech, Universite Paris-Saclay, 78026 Versailles, France; b UMR SAS, Institut Agro, INRAE, 35000 Rennes, France

**Keywords:** Soil functioning, Enzymatic activities, Organic waste products, Long-term effects

## Abstract

Land application of organic waste products (OWPs), catch crops and reduced soil tillage are accepted as sustainable management practices in agriculture. They can optimize resources by supplying nutrients to plants and helping to maintain soil fertility. They also can influence soil functions in agricultural production systems. Soil microorganisms can feed on fresh organic matter by producing extracellular enzymes. Enzyme production responds to resource availability and soil C:N:P ratios, which could limit biogeochemical cycling. Allocating resources to produce nutrient-acquiring enzymes requires a large amount of energy to achieve optimal growth. In this context, studying the use of OWPs is important, as alternatives to long-term use of mineral fertilizers, to understand the dynamics of response and how the OWPs influence production of extracellular enzymes in the soil. Effects of OWPs on soil enzymatic activities have been studied widely, but long-term effects remain poorly understood, and no information is available about differences in dynamics among systems for each biogeochemical cycle. The data described here were collected during two trials from an initial state, and they allow assessment of long-term effects of OWP application, mineral nitrogen fertilization, tillage and vegetation cover on soil enzymatic activities. Data are presented for the activities of five soil enzymes measured from 2012 to 2019: β-glucosidase, phosphatase, urease, arylamidase and arylsulfatase. Five additional enzymes were included in 2019 to supplement the analysis of biogeochemical cycles: alkaline phosphatase, phosphodiesterase, α-glucosidase, β-galactosidase and n-acetyl-glucosaminidase. These activities were measured in two trials at the EFELE study site: PROs (five OWPs applied to a corn-wheat rotation) and TS/MO (four treatments that examine interactions between OWP and type of tillage). These data can be used as a reference for future studies of soil enzymes in France and other regions (e.g. for developing reduced-tillage systems and organic or inorganic amendments, and to assess dynamics of the systems).

## Specifications Table

**Table:** 

Subject	Agricultural and Biological Sciences
Specific subject area	Soil microbiology, long-term organic systems research, soil enzymatic activity, nutrient management, crop production.
Type of data	Tables, figures, graphs, and Microsoft® Excel files
How data were acquired	The data were obtained by measuring enzymatic activities of different treatments applied in two trials. Enzymatic activities were measured according to ISO standard [1]. The methods are detailed Section 2.
Data format	Raw, analyzed
Parameters for data collection	Two long-term agronomic trials have been performed at the EFELE site since 2012: i) PROs, a randomized 4-block trial in which 5 OWP treatments are compared to a control treatment with mineral nitrogen (N) fertilization, and ii) TS/MO, a split-plot trial in which reduced vs. conventional tillage and mineral N vs. organic fertilization are studied.
Description of data collection	Soil samples have been collected from the topsoil every spring since 2012. Each sample is composed of 8 soil samples collected from each experimental plot that are homogenized and sieved to 5 mm after sampling.
Data source location	EFELE experimental site, located in Le Rheu, France (48 °06′07 N, 1 °47′44 W), and managed by INRAE, UMR SAS.
Data accessibility	Analyzed data are provided in this article. Raw data are deposited in a public repository. #Repository name: Data INRAE #Data identification number: 10.15454/KYYOPH #Direct URL to data: https://doi.org/10.15454/KYYOPH

## Value of the Data

• This dataset is based on high-frequency temporal acquisition to detail differences in dynamics enzymatic activities in agricultural soils subjected to different soil management practices.
• Communities such as agronomy, agricultural technical institutes and mathematical modellingcan benefit from these data to calibrate and design optimal agricultural practices.
• These data can be used in meta-analyses to quantify effects of repeated inputs of OWPs, tillage and crop rotations on organic matter dynamics and changes in soil quality.
• Data can be used to design statistical models to predict or evaluate effects of different treatments on soil enzymatic activities.
• Data can be used to develop a vector analysis model of enzymatic activities. This model has been suggested as a good indicator of soil resource limitation [2] and reflects microorganisms’ acquisition of C/N/P and nutrient acquisition effort.
• The data can be used by other researchers, stakeholders or organizations to quantify and model effects of repeated inputs of OWPs, tillage and crop rotations on organic matter dynamics, functioning of biogeochemical cycles (e.g. C, N, P) and changes in soil quality.

## 1 Data Description

This article includes descriptive statistics of the two trials, and figures that show effects of OWPs, catch crops, tillage and their combined effects on changes in soil enzymatic activities at the EFELE site. Data have been collected every year since the trials began in 2012 (i.e. 8 years of data currently available).

Tables show the main soil physico-chemical properties of the surface horizon in the 48 plots of the two trials at the beginning of the experiment (March 2012) (Table 1), descriptive statistics of soil activities in the PROs trial (Table 2) and TS/MO trial (Table 3), and the contents of the three dataset files (Table 4).

**Table 1:** Main soil physico-chemical properties of the surface horizon (0–25 cm for the PROs trial, 0–15 cm for the TS/MO trial) in the 48 plots of the two trials at the beginning of the experiment (March 2012).

Property	PROs trial	TS/MO trial
Clay (g 100 g^-1^)	141,6	144,6
Fine silt (g 100 g^-1^)	260,3	244,7
Coarse silt (g 100 g^-1^)	449,7	460,6
Fine sand (g 100 g^-1^)	98,7	100,0
Coarse sand (g 100 g^-1^)	49,7	50,2
Organic C (g 100 g^-1^)	1,11	1,04
Organic N (g 100 g^-1^)	0,115	0,109
C:N ratio	9,62	9,62
pH water	6,10	5,94
CEC Metson (cmol kg^-1^)	6,42	6,21
P Olsen (g 100 g^-1^)	0,019	0,014
Total P (g 100 g^-1^)	0,244	0,212

**Table 2:** Descriptive statistics of soil enzymatic activities in the PROs trial at the EFELE site for the overall trial (all data), 0 N (control without mineral fertilizer), MIN (control with mineral fertilizer) and OWP (all results of the five organic waste products with or without mineral fertilization). Enzymatic activities measured are phosphatase (PHOS), β-glucosidase (GLU), arylsulfatase (ARS), urease (URE), and arylamidase (ARN). Means and standard deviations (SD) of enzymatic activities are given in mU/g of dry soil. CV is for coefficient of variation (%).

PROs trial		PHOS	GLU	ARS	URE	ARN
Overall trial 2012–2019	*Mean* *SD* *CV* *N*	43.45 7.38 16.98 288	13.46 2.77 20.55 288	6.74 0.79 11.80 288	6.43 1.42 22.08 288	4.25 0.98 23.10 144
0N 2013–2019	*Mean* *SD* *CV* *N*	39.53 5.94 15.03 28	12.16 1.68 13.85 28	6.80 0.72 10.58 28	6.04 1.26 20.79 28	3.91 0.83 21.27 16
MIN 2013–2019	*Mean* *SD* *CV* *N*	44.91 5.82 12.95 28	13.55 2.85 21.02 28	6.57 0.83 12.66 28	5.82 1.26 21.19 28	3.84 1.05 27.23 16
OWP 2013–2019	*Mean* *SD* *CV* *N*	42.46 6.66 15.67 196	12.68 1.88 14.79 196	6.65 0.93 14.07 196	6.52 1.64 25.20 196	4.36 0.97 22.16 112

**Table 3:** Descriptive statistics of soil enzymatic activities in the TS/MO trial at the EFELE site for the overall trial (all data), CT_MIN (control with tillage and mineral fertilizer) and RT_CM (reduced tillage and cattle manure). Enzymatic activities measured are phosphatase (PHOS), β-glucosidase (GLU), arylsulfatase (ARS), urease (URE), and arylamidase (ARN). Means and standard deviations (SD) of enzymatic activities are given in mU/g of dry soil. CV is for coefficient of variation (%).

TSMO trial		PHOS	GLU	ARS	URE	ARN
Overall trial 0–15 cm 2012–2019	*Mean* *SD* *CV* *n*	47.07 7.68 16.31 96	14.21 3.15 22.21 96	6.93 0.91 13.16 96	6.93 1.92 29.90 96	4.31 1.22 28.25 48
Overall trial 15–25 cm 2012–2019	*Mean* *SD* *CV* *n*	38.79 9.53 24.57 96	11.44 3.04 26.57 96	6.70 0.74 10.98 84	5.24 1.76 33.57 96	3.14 0.75 24.04 48
CT_MIN 0–15 cm 2013–2019	*Mean* *SD* *CV* *n*	42.90 5.33 12.41 21	13.08 2.09 16.00 21	6.64 0.73 11.02 21	6.10 1.93 31.64 21	3.66 1.11 30.40 12
RT_CM 0–15 cm 2013–2019	*Mean* *SD* *CV* *n*	46.70 6.94 14.87 21	13.49 2.24 16.63 21	6.40 1.02 15.97 21	5.65 2.23 39.40 21	3.71 0.96 25.92 12

**Table 4:** Contents of the dataset files, with names and units of the variables.

File name	Content and units	Variable names
dataset_enzyme_activities.xls	Soil moisture (%)	hum
	phosphatase activity (mU *g* ^−^ ^1^ dry soil)	PHOS
	β-glucosidase activity (mU *g* ^−^ ^1^ dry soil)	GLU
	arylsulfatase activity (mU *g* ^−^ ^1^ dry soil)	ARS
	urease activity (mU *g* ^−^ ^1^ dry soil)	URE
	arylamidase activity (mU *g* ^−^ ^1^ dry soil)	ARN
	alkaline phosphatase activity (mU *g* ^−^ ^1^ dry soil)	ALP
	phosphodiesterase activity (mU *g* ^−^ ^1^ dry soil)	PDE
	α-glucosidase activity (mU *g* ^−^ ^1^ dry soil)	αGLU
	n-acetyl glucosaminidase activity (mU *g* ^−^ ^1^ dry soil)	NAG
	β-galactosidase activity (mU *g* ^−^ ^1^ dry soil)	GAL
dataset_ fertilizer.xls	Applications of mineral N (kg N ha^−1^), P (kg P_2_O_5_ ha^−1^) and K (kg K_2_O ha^−1^) fertilizers	N_mineral_fertilizer, P_2_O_5_, K_2_O
dataset_OWP.xls	Raw applications (t ha-1), contents (dry matter (g 100 g-1), ammonium (g N kg^−1^ raw product), total N (g N kg^−1^ raw product), organic matter (g OM kg^−1^ DM), organic carbon (g C kg^−1^ DM, phosphorus (g P_2_O_5_ kg^−1^ DM), potassium (g K kg^−1^ DM) and magnesium (g Mg kg^−1^ DM)) and pH	OWP_application, DM, NH_4_, total N, OM, organic C, P_2_O_5_, K, Mg and pH

**Fig. 1:**
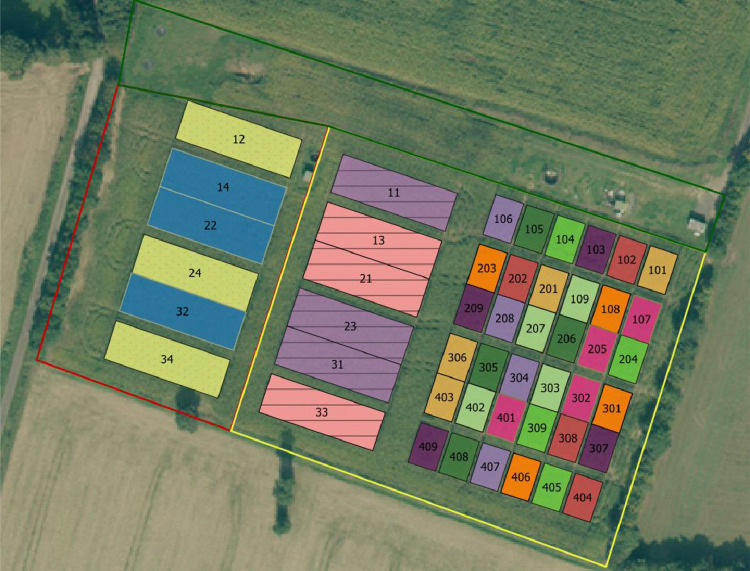
Diagram of the PROs plots (101–409) and TS/MO plots (11–34) (the zones outlined in red or yellow correspond to the reduced tillage or conventional tillage zone, respectively).

**Fig. 2:**
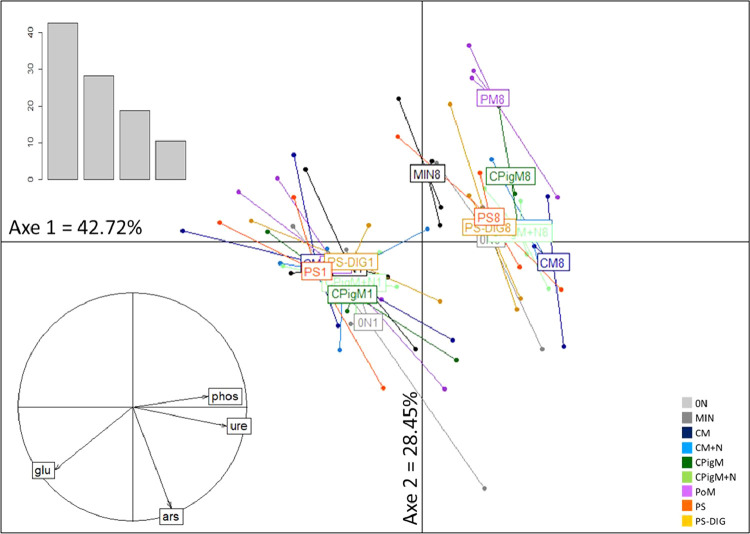
Principal component analysis of enzymatic activity in the PROs trial in 2013 (“1″) and 2019 (“8″), performed using the ade4 package of R software. (0N: control without mineral fertilizer, MIN: control with mineral fertilizer, CM: cattle manure, CM+*N*: cattle manure supplemented with mineral fertilizer, CPigM: composted pig manure, CPigM+*N*: composted pig manure supplemented with mineral fertilizer, PoM: poultry manure, PS: pig slurry and PS-DIG: digestate of pig slurry).

**Fig. 3:**
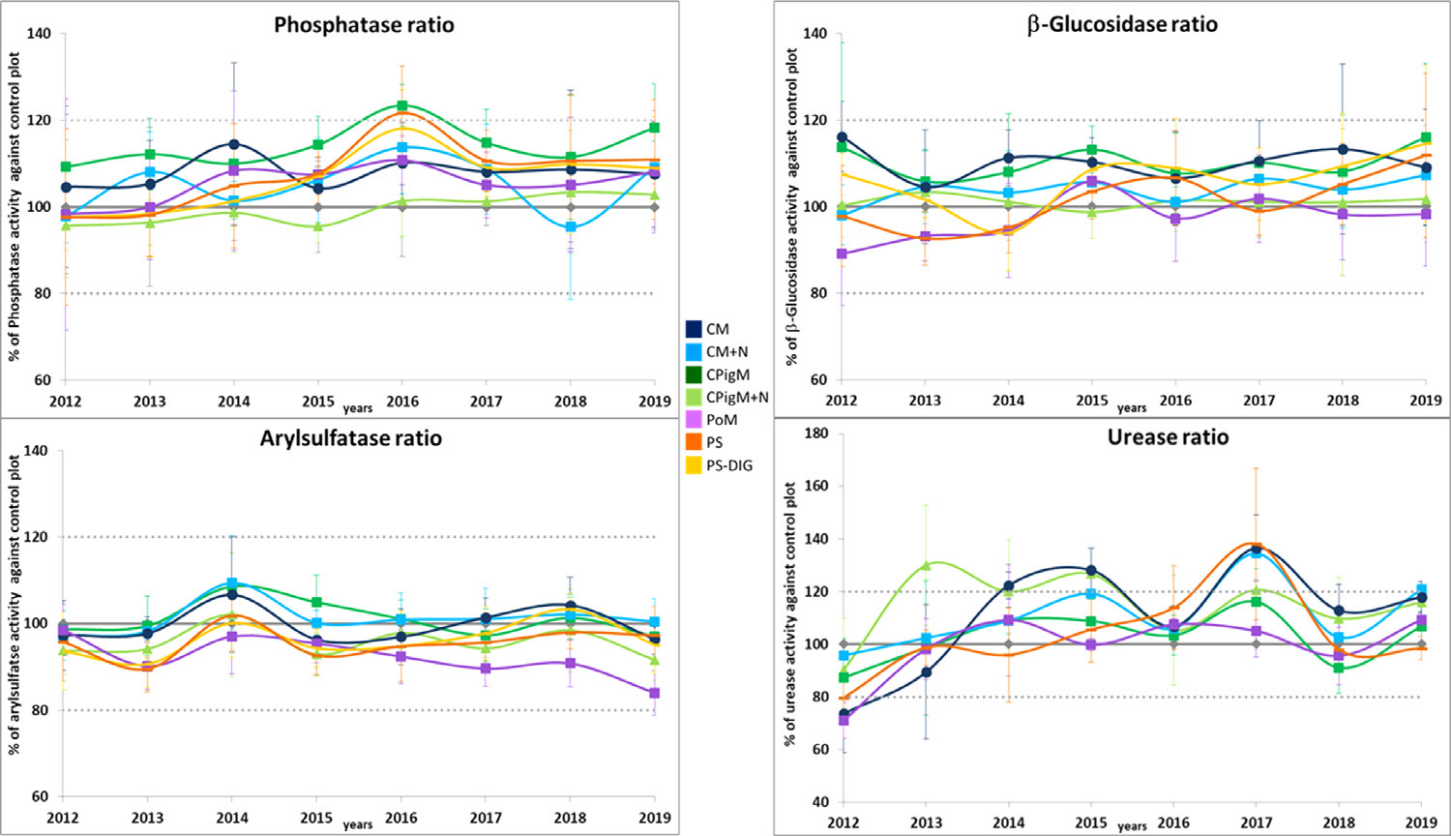
Dynamics of mean enzymatic activities of OWP plots relative to that of control plots (without fertilization) in the PROs trial from 2012 to 2019. (CM: cattle manure, CM+*N*: cattle manure supplemented with mineral fertilizer, CPigM: composted pig manure, CPigM+*N*: composted pig manure supplemented with mineral fertilizer, PoM: poultry manure, PS: pig slurry and PS-DIG: digestate of pig slurry).

**Fig. 4:**
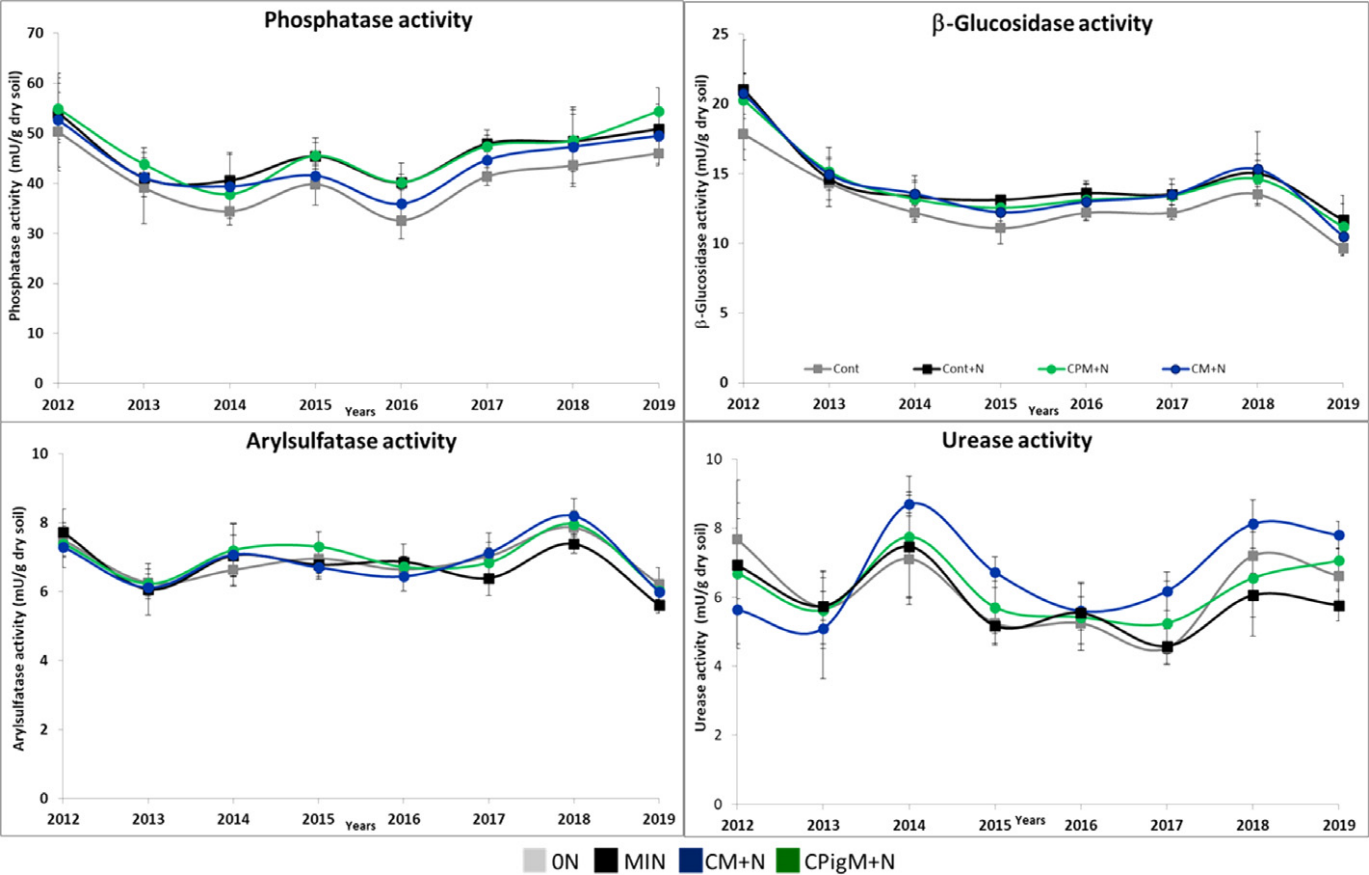
Dynamics of enzymatic activities in the PROs trial from 2012 to 2019 for four treatments: 0N: control without mineral fertilizer, MIN: control with mineral fertilizer, CM+*N*: cattle manure supplemented with mineral fertilizer and CPigM+*N*: composted pig manure supplemented with mineral fertilizer.

**Fig. 5:**
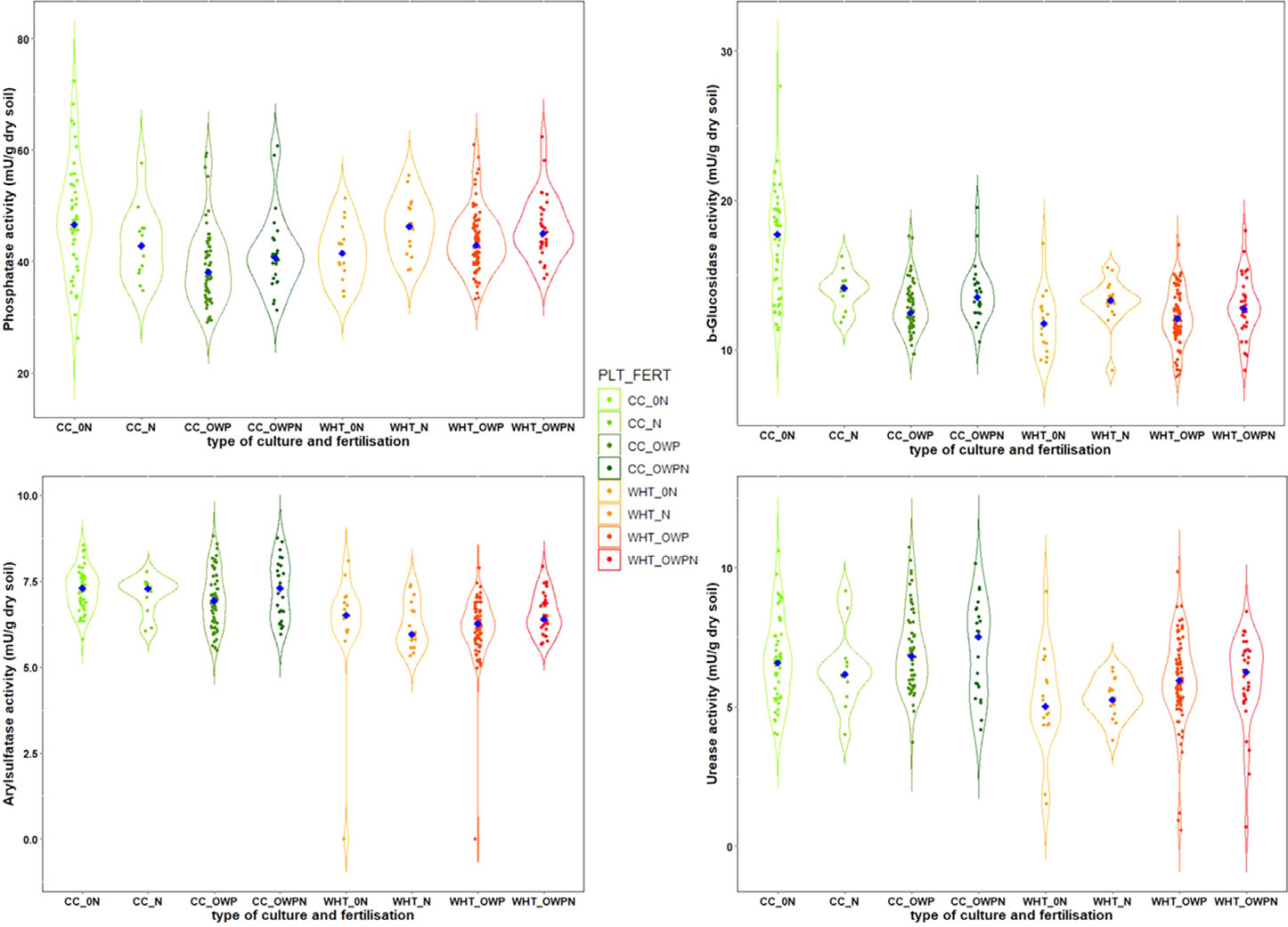
Effect of plant cover and fertilization on enzymatic activities in the PROs trial from 2012 to 2019. (CC_0N: catch crop (CC) without mineral fertilizer, CC_N: CC with mineral fertilizer, CC_OWP: CC with organic waste products (OWPs), CC_OWPN: CC with OWPs and mineral fertilizer, WHT_0N: wheat without mineral fertilizer, WHT_N: wheat with mineral fertilizer, WHT_OWP: wheat with OWPs and WHT_OWPN: wheat with OWPs and mineral fertilizer. Blue diamonds represent medians).

**Fig. 6:**
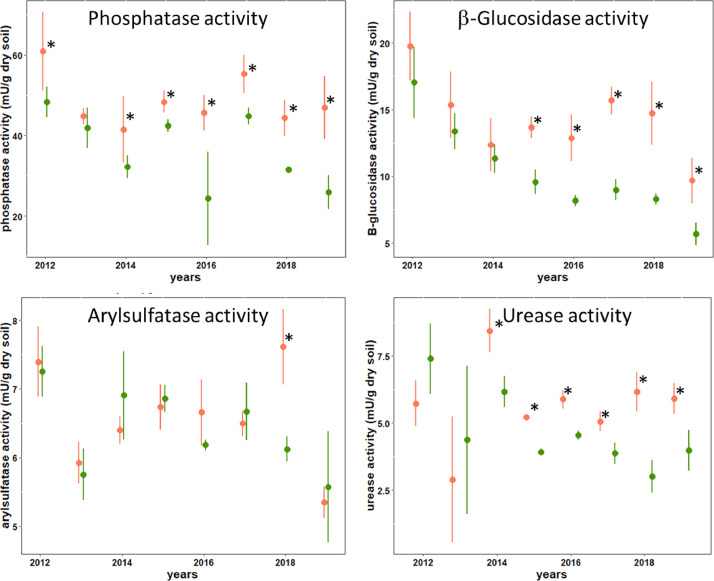
Effect of reduced tillage (RT_MIN) on enzymatic activities in the 0–15 cm (orange) and 15–25 cm (green) horizons in the TS/MO trial from 2012 to 2019. (Circles and error bars represent means and standard deviations, respectively; * is for statistical difference within year; LSD is calculated as 19–20% of difference).

**Fig. 7:**
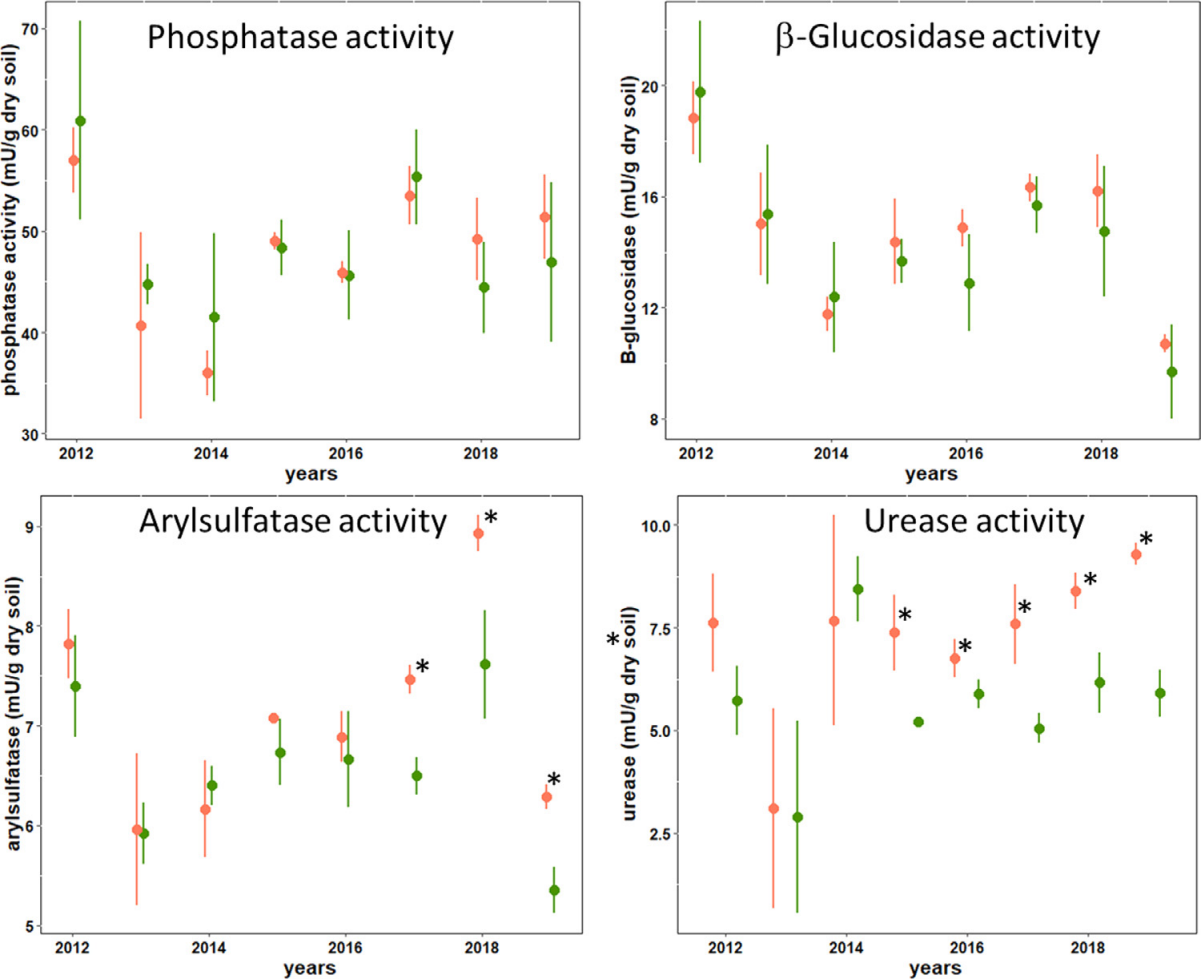
Effect of cattle manure (orange) and mineral (green) fertilization on enzymatic activities in the 0–15 cm horizon under reduced tillage in the TS/MO trial from 2012 to 2019. (Circles and error bars represent mean and standard deviation; * is for statistical difference within year; LSD is calculated as 19–20% of difference.

Figures show the map of trial plots at EFELE (Fig. 1), principal component analysis of enzymatic activities of the PROs trial in 2013 and 2019 (Fig. 2), mean enzymatic activities of OWP treatments relative to that of the unfertilized control in the PROs trial from 2012 to 2019 (Fig. 3), dynamics of enzymatic activities of four treatments in the PROs trial from 2012 to 2019 (Fig. 4), effects of plant cover and fertilization on enzymatic activities in the PROs trial from 2012 to 2019 (Fig. 5), effects of reduced tillage on enzymatic activities in both soil horizons in the TS/MO trial from 2012 to 2019 (Fig. 6) and effects of cattle manure or mineral fertilization on enzymatic activities in the 0–15 cm horizon with reduced tillage in the TS/MO trial from 2012 to 2019 (Fig. 7).

The dataset is composed of three Excel files that contain raw data (Table 4). It includes data on enzymatic activities; mineral N, P and K applications; OWP application rates and composition.

## 2 Experimental Design, Materials and Methods

### 2.1 Study site

EFELE is an experimental site (Le Rheu, France; 48 °06′07 N, 1 °47′44 W) of the SOERE PRO network (https://www6.inra.fr/valor-pro). This network is composed of 3 experimental sites in France (Qualiagro, Prospective and EFELE) and a site on Reunion Island that are designed for long-term studies of the evolution of agrosystems after repeated applications of organic residues derived from urban wastes and from animal waste that has undergone a variety of treatments (e.g. none, composting, anaerobic digestion). These experimental sites are equipped to monitor the hydrodynamic functioning of soil (TDR probes, tensiometers, temperature sensors, lysimeters) and emissions of N_2_O and CO_2_ gas (measurement chambers). The network also includes 2 trials (La Bouzule and Couhins), in which applications of organic products have been stopped for several years.

Two trials are underway at EFELE (Fig. 1):

- PROs, a complete randomized-block trial with 4 replicates, each block composed of 9 plots of 109 m^2^ each. Five OWPs were selected by combining typological criteria, such as animal species, treatment process and the reactivity of waste to its transformation in the soil. Seven OWP treatments are studied: cattle manure (CM), cattle manure supplemented with mineral nitrogen (N) fertilizer (CM+*N*), composted pig manure (CPigM), composted pig manure supplemented with mineral N fertilizer (CPigM+*N*), poultry manure (PoM), pig slurry (PS) and pig slurry digestate (PS-DIG). Two controls are included, one without mineral N fertilizer (0 N) and one with mineral N fertilizer (MIN).
- TS/MO, a split-plot trial with 3 replicates. Two tillage methods (conventional or reduced) are combined with mineral or organic fertilization. Four treatments are studied: conventional tillage and mineral N fertilizer (CT_MIN), conventional tillage and cattle manure application (CT_CM), reduced tillage and mineral N fertilizer (RT_MIN) and reduced tillage and cattle manure application (RT_MIN).

The crop rotation consists of maize (*Zea mays* L.) and winter wheat (*Triticum aestivum* L.), with a catch crop (CC) of white mustard (*Sinapis alba*) sown at the beginning of September, two months after the wheat harvest. Rates of mineral N fertilizer applied to crops were calculated using the mineral N balance-sheet method recommended in France [3]. Poultry manure (PoM), pig slurry (PS) and the digestate of pig slurry (PS-DIG) were applied in early spring every year to the growing wheat and before the sowing of maize, while cattle manure (CM) and composted pig manure (CPigM) were applied every two years before the sowing of maize. For these two treatments, the N fertilization of wheat came from mineral fertilizers.

The soil is classified as a Luvisol-Redoxisol derived from aeolian silt deposited on schist material [4]. Physical and chemical properties of the topsoil were measured at the beginning of the trials (Table 1). The climate is temperate oceanic, with a mild winter and warm summer. Mean annual rainfall is 711 mm, and mean annual temperature is 11.2 °C.

### 2.2 Soil sample collection

Soil samples have been collected every year, in early spring, from 2012 to 2019. Each sample is composed of 8 soil cores, extracted at random locations in each plot, from the 0–25 cm horizon (PROs trial) or 0–15 and 15–25 cm horizon (TS/MO trial) that are homogenized and sieved to 5 mm. The moisture content of the samples is measured after drying at 105 °C for 48 h according to [5].

### 2.3 Enzyme assays

Phosphatase (PHOS), arylsulfatase (ARS), β-glucosidase (GLU), urease (URE), arylamidase (ARN), alkalin phosphatase (ALP), phosphodiesterase (PDE), α-glucosidase (αGLU), β-galactosidase (GAL) and n-acetyl-glucosaminidase (NAG) activities are measured according to [1], derived from the respective protocols of: [6] (PHOS and ALP), [7] (ARS), [8] (URE), [9] (ARN), [10] (PDE), [11] (α/β-GLU, GAL) and [12] (NAG).

Measurements were performed on 96-well microplates (PS, Nunc, and VWR) with a Xenius reader (SAFAS, Monaco) with 4 g of soil (in triplicate) in water or buffer suspension. According to the ISO standard, PDE and ARN are incubated in Tris 50 mM pH 7.5 and ALP in Tris 50 mM pH 11. The other enzymes are incubated in distilled water. Incubations of PHOS, ARS, GLU, ALP, PDE, αGLU, GAL and NAG are performed at 37 °C. Soil suspensions are incubated with substrates: 4-nitrophenylphosphate for 30 min for PHOS and PAK, 4-nitrophenyl sulfate for 4 h for ARS, 4-nitrophenyl β-glucopyranoside for 1 h for GLU, bis-nitrophenylphosphate for 1 h for PDE, 4-nitrophenyl α-glucopyranoside for 1 h for αGLU, 4-nitrophenyl β-galactopyranoside for 3 h for GAL, and 4-nitrophenyl N-acetyl-β-d-glucosaminide for 2 h for NAG. One well per triplicate is incubated without substrate for soil controls. The reaction is stopped with CaCl_2_ and Tris pH 12. URE activity is quantified by mixing a soil solution with urea (tests) or water (controls) and incubating it for 3 h at 25 °C. The quantification of NH4^+^ is achieved by adding ammonium salicylate and ammonium cyanurate. For ARN, the soil solution is incubated with l- leucine β- naphthylamide for 2 h, and the reaction is stopped with ethanol. The β-naphthylamine produced is colored with p-(dimethylamino)cinnamaldehyde (DMCA).

The concentration of the product released is reported to a range of para-nitrophenol (for PHOS, ARS, GLU, ALP, PDE, αGLU, GAL and NAG), NH_4_Cl (for URE) or β-naphthylamine (for ARN). Enzymatic activities are calculated and expressed in mU (nanomole equivalent of product released per min) per g of dry soil.

## Ethics Statement

Not applicable

## CRediT Author Statement

**N.Cheviron:** Conceptualization, Methodology, Validation, Formal analysis, Investigation, Data Curation, Writing – Original Draft, Writing-Review and Editing, visualisation, Supervision, Project Administration; **I. Amadou:** Formal analysis, Investigation, Data Curation, Writing – Original Draft; **V Grondin:** Methodology; **C Marrauld:** Methodology; **C Mougin:** Writing – Original Draft; **T. Morvan**: Writing – Original Draft, Writing-Review and Editing, Project Administration, Funding acquisition

## Declaration of Competing Interest

The authors declare that they have no known competing financial interests or personal relationships that could have appeared to influence the work reported in this article.
